# Genome sequences of *Mycobacterium* sp. Elmwood and accompanying phage, isolated from a public swimming pool in Nebraska

**DOI:** 10.1128/mra.00896-24

**Published:** 2024-09-30

**Authors:** Daniela Hernandez-Velazquez, Madelynn K. Vasquez, Rana Petre, John A. Kyndt

**Affiliations:** 1 Universidad Veracruzana, Xalapa, Veracruz, Mexico; 2 College of Science and Technology, Bellevue University, Bellevue, Nebraska, USA; 3 Erasmus Brussels University of Applied Sciences and Art, Brussels, Belgium; Department of Biology, Queens College, Queens, New York, USA

**Keywords:** *Mycobacterium*, non-tuberculosis, swimming pool, bacteriophage, antibiotic resistance, anti-phage defense

## Abstract

Genome sequencing of a non-tuberculosis *Mycobacterium* species, isolated from a public pool, shows that the genome contains several genes for antibiotic resistance and anti-phage defense, which are absent from other related *Mycobacteria*. Metagenomic binning also provided the genome of the accompanying phage, which is distinct from other mycobacterial phages.

## ANNOUNCEMENT

Nontuberculous mycobacteria (NTM), also known as environmental or atypical mycobacteria, are mycobacteria that do not cause tuberculosis or leprosy/Hansen’s disease (1–3). However, several NTM can cause pulmonary diseases that resemble tuberculosis and are an emerging healthcare-acquired infection, found in environmental systems and water heating and cooling systems (4–6). The FDA and CDC have an ongoing warning for NTM and their potential harmful effects on individuals who are older, immunocompromised, or have other medical conditions like open wounds (7–11).

Microbial samples were collected from the Elmwood public pool in Omaha, NE, USA (41.25494 N, 96.00510 W), in July 2023, as part of a project testing for microbial and algal contamination in pools. Samples were collected in sterile 50-mL tubes from the deep end of the pool and stored at 4°C overnight. A volume of 6 mL was used for genomic DNA extraction using the Qiagen DNeasy Kit. DNA analysis using QuBit and NanoDrop showed an *A*_260/280_ ratio of 1.90. The sequencing libraries were prepared using the Illumina DNA Library Prep Kit and sequenced by an Illumina MiniSeq using 500 µL of a 1.8 pM library. Paired-end (2 × 150 bp) sequencing generated 920,658 reads and 139 Mbp. Quality control of the reads was performed using FASTQC (version 1.0.0) within BaseSpace (Illumina), using a k-mer size of 5 and contamination filtering. Illumina adapter sequences were removed using the FASTQ Toolkit (version 2.2.6) within BaseSpace (Illumina). The genome was assembled using the Metagenomic binning function in BV-BRC (12, 13), which uses MetaSPAdes (version 3.12.0) (14). This provided a single bacterial genome of 6,842,446 bp and 67.0% GC. The genome characteristics are summarized in Table 1. The final genome was 99.9% complete according to CheckM (version 1.1.6) (15) and was annotated by NCBI PGAP (version 6.6) (16). Default parameters were used for all software applications unless otherwise noted.

**TABLE 1:** Overview of genome features of the genomes of *Mycobacterium* species related to *Mycobacterium* sp. Elmwood^*a*^

Species	Size (Mb)	% GC	Coverage	Contigs	*N*_50_	CDS	tRNAs	dDDH %	ANI %	Accession	Habitat	Country, state
												
*Mycobacterium* sp. Elmwood	6.8	67	21×	249	55,081	6,728	46	–	–	JBFUXV000000000	Swimming pool	USA, NE
*Mycobacterium adipatum* YC-RL4	6.1	67.4	N/A^*b*^	2	5,801,417	5,912	47	86.7	97.5	CP015596.1	Farmland	China, Shandong
*Mycobacterium* sp. MBM	6.7	66.5	10×	274	194,677	6,734	50	35	86.6	JAKJHY000000000	Coastal soil	India, West Bengal
*Mycobacterium frederiksbergense* LB 501T	6.7	67.1	194×	4	6,086,872	6,697	46	32.4	85.8	CP038799.1	Soil	Belgium
*Mycobacterium* sp. P9-22	6.8	66.9	275×	38	608,156	6,633	47	31.1	84.6	NPKP00000000	Fungal root	Austria, Lower Austria

^*a*^ ANI percentage is based on bidirectional ANIb values for strain Elmwood, calculated using JSpecies (17). dDDH was determined using the Type (Strain) Genome Server provided by DSMZ (https://tygs.dsmz.de) (18).

^*b*^ N/A is not available.

**Fig 1:**
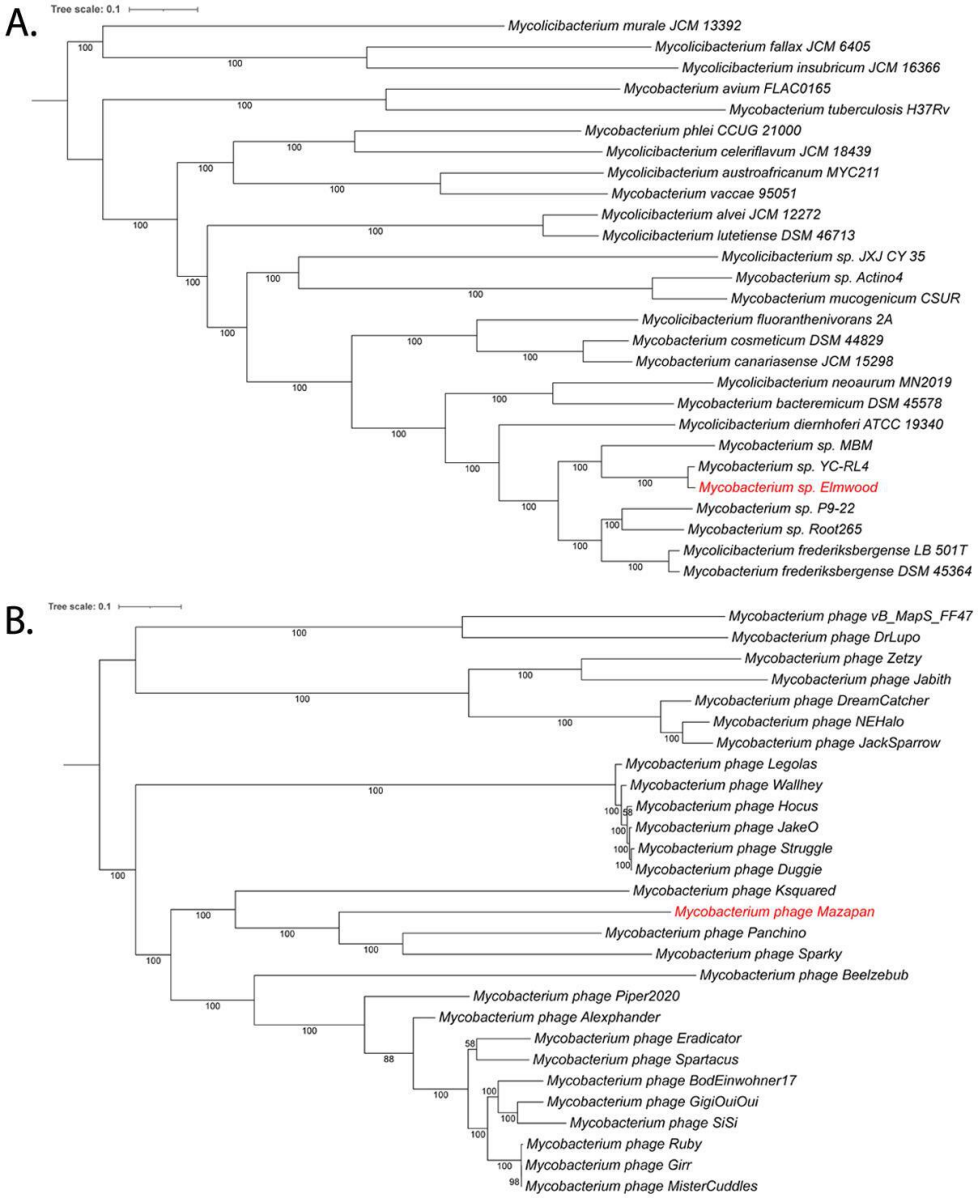
Whole-genome-based phylogenetic tree of *Mycobacterium* compared to representative and closely related genomes (**A**) and of the *Mycobacterium* phage to other phages from North American isolates (**B**). The phylogenetic trees were generated using the codon tree method within BV-BRC (12), which used PGFams as homology groups. A total of 754 PGFams were found in the WGS analysis, and the aligned proteins and coding DNA from single-copy genes were used for RAxML analysis (19, 20). One hundred rounds of the “Rapid bootstrapping” option of RaxML were used to generate the support values for the phylogenetic trees. The branch length tree scale is defined as the mean number of substitutions per site, which is an average across both nucleotide and amino acid changes. New genomes are in red. iTOL was used for tree visualization (22).

Whole-genome-based phylogenetic analysis was performed using RAxML (19, 20). The closest relatives were identified using the Similar Genome Finder feature that uses Mash/MinHash (version 2.3) in BV-BRC (21). These analyses placed the new genome on a separate clade with strain YC-RL4 isolated from China (Fig. 1A). A JSpecies comparison (version 4.1.1) (17) of average percentage nucleotide identity showed 97.5% ANI to YC-RL4, but all other strains are far below the arbitrary species cutoff (95%) (Table 1), which indicates a species differentiation of this clade. dDDH analysis provides a similar pattern as the ANI comparison (Table 1). In addition, the Metagenomic binning (13) showed the presence of a complete phage (57 kbp) that is distinct from other sequenced North American mycobacterial phages, as shown in the phylogenetic phage tree in Fig. 1B.

A comparative systems analysis in BV-BRC (23–25) of strain Elmwood to the other mycobacteria revealed 91 unique PGFams. Besides several phage genes, the genome also contains a unique predicted YdcE-YdcD toxin/antitoxin system (programmed cell death system), which has recently been shown to be involved in anti-phage defense (26), consistent with the presence of the mycobacterial phage in this strain. Antibiotic resistance in *Mycobacteria* is of growing concern (27–29). It is therefore noteworthy that the new genome contains predicted beta-lactamase and tetracycline resistance genes (besides >50 other AMR targets). The presence of *Mycobacteria* in the pool itself is not surprising, given their known resistance to chlorine (30); however, the presence of multiple resistance genes and a unique phage system indicates the need for further studies of this novel isolate.

## Data Availability

This Whole Genome Shotgun project has been deposited at DDBJ/ENA/GenBank with the following accession number JBFUXV000000000. The phage genome has been submitted to GenBank with accession number PQ115172. The raw sequencing reads have been submitted to SRA and the corresponding accession number is SRR29988839.
